# Computed tomography: an efficient, opportunistic method for assessing body composition and predicting adverse outcomes in cancer patients

**DOI:** 10.1590/0100-3984.2023.56.6e3-en

**Published:** 2023

**Authors:** Adriano de Araújo Lima Liguori, Ana Paula Trussardi Fayh

**Affiliations:** 1 Radiologist for the Liga Norte Riograndense Contra o Câncer, Professor of Radiology at the Universidade Federal do Rio Grande do Norte (UFRN), Natal, RN, Brazil. Email: adrianoliguori@gmail.com; 2 Associate Professor III in Nutrition at the Universidade Federal do Rio Grande do Norte (UFRN), Natal, RN, Brazil

In recent years, we have seen an increase in the use of biomarkers, including those related to body composition, in the field of radiology. In that context, computed tomography (CT) and magnetic resonance imaging are considered the gold standards for evaluating body composition^(1,2)^. Specifically on CT image analysis, it is possible to determine the mass of the internal organs and to differentiate among specific tissues, such as visceral adipose tissue, subcutaneous adipose tissue, and muscle groups. In addition, CT image analysis can provide information on skeletal muscle mass (SMM) and skeletal muscle density (SMD), which are closely related to fatty infiltration of the muscle, and their impact on muscle function^(3,4)^. Therefore, CT imaging has been widely used as an opportunistic method for assessing body composition in diseases for which imaging examinations are performed for diagnosis and follow-up, such as cancer^(5,6,7)^.

In the literature, there is robust evidence of an association between low SMD and postoperative complications in patients with various types of cancer^(8,9,10,11,12)^. However, studies of patients with renal cancer are still scarce. One systematic review and meta-analysis that included 28 studies, with a collective total of 6,608 patients with renal cell carcinoma (RCC), sought to identify associations between body composition and clinical outcomes^(13)^. Although the authors found that low SMM and low SMD were associated with higher overall mortality, the heterogeneity of the studies made it impossible to perform a meta-analysis focusing on perioperative outcomes. That demonstrates the importance of carrying out new studies seeking to investigate the effect that muscle characteristics (e.g., SMM and SMD) on surgical outcomes in this population, as was done in the study entitled “Impact of preoperative body composition in patients with renal cell carcinoma submitted to surgical treatment”, conducted by Carniatto et al.^(14)^ and published in this issue of **Radiologia Brasileira**. The authors evaluated the impact of preoperative body composition in patients undergoing surgical treatment for RCC. Their study was retrospective, including 52 patients with RCCs, the majority of which were of the clear cell subtype. Although they found no association between preoperative body composition and the frequency of perioperative complications in patients undergoing partial or total nephrectomy, they did find that the skeletal muscle gauge (the product of the SMM index and the SMD) was associated with the length of hospital stay and with overall survival.

The main limitations of the Carniatto et al.^(14)^ study are its retrospective methodology, its small sample size, and the fact that the statistical analysis did not reveal a significant association between preoperative body composition and perioperative complications. To delve deeper into this field, it is expected that prospective studies involving larger samples of patients with clear cell RCC will provide additional information.

There are some obstacles to the widespread application of knowledge in the field of body composition in daily radiology practice, such as limited access to specific software and a lack of remuneration for the time dedicated to post-processing. As a solution and future perspective, we hope that artificial intelligence algorithms^(15)^, especially those dedicated to automated or semi-automated segmentation, will be increasingly incorporated into work routines, which could allow the definitive use of body composition data and their inclusion in radiology reports.
